# Evaluation and Obstacle Analysis of Emergency Response Capability in China

**DOI:** 10.3390/ijerph191610200

**Published:** 2022-08-17

**Authors:** Huiquan Wang, Hong Ye, Lu Liu, Jixia Li

**Affiliations:** 1 School of Politics and Public Administration, China University of Political Science and Law, Beijing 100088, China; 2 School of Foreign Studies, China University of Political Science and Law, Beijing 100088, China; 3 School of Engineering and Technology, China University of Geosciences (Beijing), Beijing 100083, China; 4 School of Government, Beijing Normal University, Beijing 100875, China

**Keywords:** Chinese provinces and municipalities, emergency response capacity, evaluation system, entropy method, obstacle degree model

## Abstract

Emergency response capability evaluation is an essential means to strengthen emergency response capacity-building and improve the level of government administration. Based on the whole life cycle of emergency management, the emergency capability evaluation index system is constructed from four aspects: prevention and emergency preparedness, monitoring and early warning, emergency response and rescue, and recovery and reconstruction. Firstly, the entropy method is applied to measure the emergency response capability level of 31 Chinese provinces from 2011 to 2020. Second, the Theil index and ESDA (Exploratory Spatial Data Analysis) are applied in exploring the regional differences and spatial-temporal distribution characteristics of China’s emergency response capacity. Finally, the obstacle degree model is used to explore the obstacle factors and obstacle degrees that affect the emergency response capability. The results show that: (1) The average value of China’s emergency response capacity is 0.277, with a steady growth trend and a gradient distribution of “high in the east, low in the west, and average in center and northeast” in the four major regions. (2) From the perspective of spatial distribution characteristics, the unbalanced regional development leads to the obvious aggregation effect of “high-efficiency aggregation and low-efficiency aggregation”, and the interaction of the “centripetal effect” and “centrifugal effect” finally forms the spatial clustering result of emergency response capability level in China. (3) Examining the source of regional differences, inter-regional differences are the decisive factor affecting the overall differences in emergency response capability, and the inter-regional differences show a reciprocating fluctuation of narrowing–widening–narrowing from 2011 to 2020. (4) Main obstacles restricting the improvement of China’s emergency response capabilities are “the business volume of postal and telecommunication services per capita”, “the daily disposal capacity of city sewage” and “the general public budget revenue by region”. The extent of the obstacles’ impacts in 2020 are 12.19%, 7.48%, and 7.08%, respectively. Based on the evaluation results, the following countermeasures are proposed: to realize the balance of each stage of emergency management during the holistic process; to strengthen emergency coordination and balanced regional development; and to implement precise measures to make up for the shortcomings of emergency response capabilities.

## 1. Introduction

Emergencies refer to events that are random in time, multiple in inducing cause, public in scope, and pose significant threats and damages to the safety of life and property of citizens, public order, regional or national security, and are usually classified into the following four types: natural disasters, accident disasters, public health events, and social security events [1]. As emergencies are often unpredictable and can easily trigger a chain reaction of various industries and groups in society, they pose a great threat to people’s health, life and property safety, stable social and economic development, and the implementation of national strategic planning. A series of emergencies such as the “9/11 terrorist attack”, the “3/11 great east Japan earthquake”, and the “COVID-19 pandemic” have shown that the modernization of society has not reduced the risk of emergencies, but rather increased their likelihood.

Under the wave of globalization with social, economic, information, and technological integration, effective control of the catastrophic effects of emergencies is an important issue of great concern to all countries in order to minimize the impact of social unrest, economic stagnation, and health infringement on the public, society, the country, and the world. How can we deal with emergencies in a scientific and rational way? How can we eliminate the serious consequences of emergencies and how effective is the implementation? Solving these problems has become the key to improving emergency management capabilities. Therefore, evaluating the emergency response capability of current emergencies based on objective indicators and optimizing the emergency management system for deficiencies to achieve effective disposal of disasters has always been the focus and difficulty of political and academic circles, which also has strong guiding significance for the systematization, standardization, rule of law and normalization of emergency management.

Since the turn of the millennium, China’s economic, social, and natural environment has entered a phase when various types of emergencies occur with high probability, great impact and destruction. As German sociologist Ulrich Beck said about “risk society” [2], from the “SARS crisis” in 2002 and the “Wenchuan earthquake” in 2008, to the “3/1 Yunnan Kunming train station violence and terrorism case” in 2014, the “8/12 Tianjin Binhai New Area explosion” in 2015, and the “COVID-19 pandemic” in 2020, the frequency, impact and damage caused by various emergencies in China have been increasing. According to statistics, China’s average annual abnormal death toll caused by natural disasters, accident disasters, public health, and social security emergencies is more than 200,000, disability more than 2 million, and economic losses as high as $97 billion, accounting for about 6% of the country’s total GDP [3]. Therefore, it is necessary to conduct an in-depth study on the evaluation of China’s emergency response capacity. In view of this, this paper takes 31 provinces (cities) in China from 2011 to 2020 as the research objects, adopts the entropy method, Theil index, and ESDA (Exploratory Spatial Data Analysis) to evaluate the emergency response capability and its spatial-temporal differentiation pattern, and explores obstacle factors that restrict the improvement of emergency capacity by means of a barrier degree model, in order to provide policy basis and experience reference for building emergency response capacity in China.

## 2. Literature Review

Emergency response capacity is the ability of the government to take measures to successfully mitigate the effects of natural disasters. This definition was proposed by the North Carolina Emergency Management Branch of the United States in its Local Hazard Mitigation Plan Manual, which has been internationally accepted [4]. On this basis, Han Z believes that emergency response capability is the ability of the government to predict, monitor, control, and coordinate emergencies that may occur or have occurred in order to perform emergency management functions [5]. Liu C and Ma Z consider emergency response capability as the cultivation and enhancement of disaster prevention and mitigation capabilities of the state or social institutions in terms of human resources, science and technology, organization, and resources [6,7]. Evaluating the country’s emergency response capability can identify its deficiencies and weaknesses, promote the improvement of emergency management legal systems, institutions, and mechanisms, and is of great significance to improve emergency management. However, how to evaluate emergency response capacity scientifically is a major problem faced by most countries in the world, and this has attracted widespread attention from government and academic circles. Therefore, this paper focuses on two aspects of government practice and scholars’ research to analyze the relevant studies.

In terms of government practice, the United States was the first country to carry out emergency response capacity assessment, and the Federal Emergency Management Agency (FEMA) and the National Emergency Management Association (NEMA) jointly studied a system of Capability Assessment for Readiness CAR (CAR) for state and local governments in 1997, which focused on 56 elements, 209 attributes, and 1014 indicators generated from 13 management functions of emergency management subjects [8,9]. Japan is also relatively advanced in researching emergency response capacity assessment and set up an evaluation item of disaster prevention capacity of local public organizations in 2002, including nine aspects such as crisis mastery and assessment, countermeasures to mitigate danger, rectification system, and information and communication system [10,11]. Australia first appointed a high-level panel of senior officials through a government committee in 2001 to review current national natural disaster management practices and made 12 targeted recommendations for reform [12]. In China, the Second Standing Committee of the 10th National Committee of the Chinese People’s Political Consultative Conference (CPPCC), held in Beijing in March 2004, pointed out that the basic work of developing and implementing a “disaster capacity evaluation index system” should be accelerated [13]. In addition, Canada and the United Kingdom have also achieved certain results in emergency response capacity assessment [14,15].

Academic studies on the evaluation of emergency response capacity broadly include two aspects, one of which is the evaluation of the whole regional system. For example, Nirmal et al. [16], Rahayu et al. [17], Christofer et al. [18], Boeriu [19], Bharadwaj et al. [20], and Bojan et al. [21] have evaluated the emergency response capacity of regional systems such as countries, coastal cities, university campuses, medical institutions, highways, and enterprises, respectively. In addition, some other scholars have evaluated the emergency response capacity in China and some regions. Kong and Sun took COVID-19 epidemic prevention and control in rural areas as an example and proposed relevant suggestions in terms of strengthening rural psychological interventions and expanding the scope of emergency management subjects [22]. Chen M et al. constructed 25 indicators for emergency response capacity assessment through target level, criteria level, and sub-criteria level, based on Analytic Hierarchy Process (AHP) and Fuzzy Comprehensive Evaluation (FCE) to determine the weights of the indicators as well as to analyze the decision-making issues, and verified the effectiveness of the established system by taking a fire station in Zhengzhou City as an example [23]. The second is the evaluation of emergency management subjects. For example, Elhadi et al. measured physicians’ and nurses’ knowledge and preparedness for COVID-19 in a study of 2000 health care workers in 21 hospitals in Libya [24]. Goniewicz et al. evaluated the emergency collaboration capacity of EU member states to purchase medical supplies during the COVID-19 pandemic and proposed that the government should shift from an isolated decision-making method to accepting multidisciplinary and interdisciplinary cooperation [25]. Ma Z et al. constructed a Tobit Model to analyze community resilience and residents’ emergency preparedness using survey data from 327 households in four districts and counties affected by the Wenchuan and Lushan earthquakes in Sichuan Province, China [26]. 

To sum up, scholars at home and abroad have yielded relatively fruitful results on emergency response capacity, but there are still two problems that need to be solved urgently. On the one hand, the studies focus too much on the establishment of indicator systems and are mostly qualitative and single evaluation, such as county-level CDCs and municipal public hospitals, while there are fewer studies for national emergency management capacity assessment, and global horizontal comparisons are even rarer. On the other hand, the emergency management process is fragmented, and the capacity evaluation objects are set as a number of discontinuous activities that severed the holistic process of emergency management capacity. Although most of the evaluation index systems consider the influencing factors in a comprehensive way, they do not rationalize the logical sequence of the emergency management process, and the logical relationships between levels do not appear adequately clear and lack a structured system. Therefore, compared with existing studies, the contributions of this paper are mainly the following: (1) In terms of the research object, it is the first time that a comprehensive and systematic evaluation of emergency response capability, spatial and temporal distribution characteristics, and obstacle factors in China is conducted. (2) In terms of research content, this paper explores emergency response capacity with a new evaluation model where the emergency response process is evaluated. Also, the establishment of indicators focuses on the availability and quantification of data to reflect China’s emergency response capacity more objectively and accurately. (3) In terms of research methods, the improved Entropy Weight Method with time variables is combined with the Obstacle Degree Model to solve the interference of human factors in subjective weighting evaluation methods such as AHP and the Delphi Method, which is a major innovation in the current research method of emergency capacity evaluation.

## 3. Method and Data

### 3.1. Entropy Weight Method

The Entropy Weight Method is an objective assignment method, which determines the weight of each index by dimensionless processing of data and then combining entropy weights [27]. Since the time span of the data used in this study is ten years, an attempt was made to add a time variable to the traditional entropy method to evaluate the research object more comprehensively and accurately through improvement. The specific steps are as follows.

① Construct the original indicator matrix data. If there are h years, n provinces (cities) and m evaluation indicators, then the original indicator matrix is as follows:

(1) $$X=\left\{x_{hij}\right\}h\cdot n\cdot m,\left(1\leq i\leq n,1\leq j\leq m\right)$$

In Equation (1), $x_{hij}\;$ is the value of the j-th indicator for province (city) i in year h. In this study, h, n and m are 10, 31 and 30, respectively. 

② Standardize the indicators to unify the data of different dimensions and orders of magnitude.

When the indicators are positively correlated with the evaluation results, Formula (2) is adopted, and the larger the value after processing, the greater the improvement effect on the evaluation layer:

(2) $$Y_{ij}=\frac{x_{ij}-\min\left(x_{i}\right)}{\max\left(x_{j}\right)-\min\left(x_{j}\right)}$$

Equation (3) is used to standardize the negative indicators, and the larger the value after treatment, the greater the hindering effect on the evaluation layer:

(3) $$Y_{ij}=\frac{\max\left(x_{j}\right)-x_{ij}}{\max\left(x_{j}\right)-\min\left(x_{j}\right)}$$

③ Calculate the proportion of the i-th province (city) in the indicator under the j-th indicator after standardization, and its formula is:

(4) $$P_{ij}=\frac{x_{ij}}{\sum_{i=1}^{n}x_{ij}}\;(i\;=1,\;2,\;\ldots ,\;n;\;j=1,\;2,\;\ldots ,\;m)$$

④ The information entropy value $E_{j}$ of the j-th indicator is shown in Equation (5), where k is the reciprocal of ln(n), and *p_ij_* is the weight of the indicator calculated in Equation (4):

(5) $$E_{j}\;=-k\sum_{i=1}^{n}p_{ij}\ln\left(P_{ij}\right),\;k\;>\;0,\;k\;=\frac{1}{\ln(n)},\;E_{i}\;\geq \;0$$

⑤ Calculate the redundancy (coefficient of variation) of the entropy value of each indicator. For j indicators, the greater the variation of the indicator value $x_{ij}$, the greater the evaluation effect on the program and the smaller the entropy value:

(6) $$D_{j}\;=1-E_{j}$$

⑥ Calculate the weights $W_{j}$ for the corresponding indicators:

(7) $$W_{j}=\frac{D_{j}}{\sum_{j=1}^{m}D_{j}}\;(j\;=\;1,\;2,\;\ldots ,\;m)$$

⑦ Measure the comprehensive score of emergency ability $S_{j}$:

(8) $$S_{j}=\sum_{j=1}^{m}W_{j}\cdot P_{ij}\;(j\;=\;1,\;2,\;\ldots ,\;m)$$

### 3.2. Theil Index Model

The Thiel index, named by Thiel’s use of the concept of entropy in information theory to calculate income inequality, has been widely used by academics to compare different dimensions of various regions and analyze the sources of their differences because of the way it is defined to calculate the differences of individuals in the system [28,29]. This paper constructs the overall Theil index of the difference in emergency response capacity among 31 provinces (cities) in China, which can be expressed by the following formula:

(9) $$T\;=\frac{1}{n}\sum_{i=1}^{n}\frac{y_{i}}{\bar{y}}\log\left(\frac{y_{i}}{\bar{y}}\right)$$

(10) $$T_{w}\;=\sum_{k=1}^{m}\left(\frac{n_{k}}{n}\frac{\bar{y_{k}}}{\bar{y}}\right)TL_{k}$$

(11) $$T_{b}=\sum_{k=1}^{m}\frac{n_{k}}{n}\left(\frac{\bar{y_{k}}}{\bar{y}}\right)\log\left(\frac{\bar{y_{k}}}{\bar{y}}\right)$$

In the above equation, T is the total Thiel index of emergency response capacity, n is the number of provinces (cities), $y_{i}$ denotes the level of emergency response capacity of the i-th province (city), $\bar{y}$ is the average level. When the emergency response level of each province is absolutely balanced, i.e., when $y_{i}\;$= $\bar{y}$, it can be deduced from Equation (9) that T=0. The closer the value of the Thiel index is to 0, the smaller the inter-individual difference is, on the contrary, the greater the difference is. The 31 provinces (cities) in China are divided into several regions, and thus the total Thiel index is decomposed into intra-regional differences $T_{w}$ (Equation (10)) and inter-regional differences $T_{b}$ (Equation (11)), where m is the number of regions and $n_{k}$ denotes the number of provinces contained in the k-th region. On this basis, the ratio of inter-regional and intra-regional differences to total differences is calculated respectively, which is called the contribution rate to the overall difference: the contribution rate of intra-regional differences is $T_{w}$/T, and the contribution rate of inter-regional differences is $T_{b}$/ T.

### 3.3. Exploratory Spatial Data Analysis

Exploratory Spatial Data Analysis (ESDA) is based on statistical principles and visualization techniques such as maps to combine data with geospatial information, aiming to reveal the spatial distribution among research objects and discover spatial association patterns [30].

(1) Global spatial autocorrelation

The global spatial autocorrelation analysis can measure the degree of spatial association and spatial differences in the overall level of emergency response capacity of each province, and the commonly used statistical indicators include Moran’s I [31], Geary’s C [32], and General G [33], etc. Among them, Moran’s *I* was proposed by Moran in 1948, which reflects the degree of similarity of attribute values of spatially adjacent regional units, and this index is often used to detect the spatial distribution characteristics of the whole study area, and its formula is:

(12) $$Moran’s\;I=\frac{\sum_{i=1}^{n}\sum_{j\neq 1}^{n}W_{ij}\left(x_{i}-\bar{x}\right)}{S^{2}\sum_{i=1}^{n}\sum_{j=1}^{n}W_{ij}x_{i}x_{j}/\sum_{i=1}^{n}\sum_{j=1}^{n}x_{i}x_{j}}$$

In Equation (12), I is the Moran index, $S^{2}=\sum_{i=1}^{n}{\left(x_{i}-\bar{x}\right)}^{2}$, n is the total number of study provinces, $x_{i}$ and $x_{j}$ are the observations of the attribute feature on provinces i and j, $W_{ij}$ is the normalized spatial weight matrix, and $\bar{x}$ is the average of all observations of the attribute feature x in the n study regions.

(2) Local spatial autocorrelation

The global Moran’s I assumes that the space is homogeneous [34], which is deficient in that it does not reflect what kind of correlation the local regions have and the degree of correlation [35]. Therefore, Anselin defined Local Indicators of Spatial Association (LISA) to measure the significant degree of spatial aggregation between each regional unit and the attribute values of its surrounding regions [36]. The local Moran’s I as LISA is a localized version of the global Moran’s I statistic, which is defined as:

(13) $$Moran’s\;I_{i}=\frac{x_{i}-\bar{x}}{S}\sum_{j=1}^{n}\bar{W_{ij}}\left(x_{i}-\bar{x}\right)$$

In Equation (13), n, $x_{i}$, $\bar{x}$, $W_{ij}$ have the same meaning as Formula (12); if the local Moran’s I index is significantly positive, it indicates that province i in the local space and its neighboring provinces present spatial agglomeration with similarity (high-high (HH) or low-low (LL)) in the level of emergency response capacity; if the Moran’s I index is significantly negative, it indicates spatial agglomeration with non-similarity (high-low (HL) or low-high (LH)). 

### 3.4. Obstacle Degree Model

In the evaluation process of emergency response capability in China, it is not only necessary to measure its level and spatial and temporal distribution, but a more practically significant issue is to understand the hindering factors for the improvement of emergency response capability in different regions and gradually clarify the shortcomings that restrict the development of each region [37,38]. Therefore, this study introduces the Obstacle Degree Model to explore the obstacle factors and obstacle degree that affects emergency response capacity. The specific calculation formula is as follows:

(14) $$O_{ij}=1-x_{ij}$$

(15) $$I_{j}=\frac{O_{ij}\cdot w_{j}}{\sum_{j=1}^{n}O_{ij}\cdot w_{j}}$$

(16) $$U=\sum I_{j}$$

In the formula, $O_{ij}$ indicates the deviation degree of the j-th indicator of the i-th region, i.e., the gap between the single indicator and the maximum target; $w_{j}$ indicates the factor contribution degree, that is, the degree of contribution of the single indicator to the total target, and is the weight value of the j-th indicator; $I_{j}$ is the obstacle degree, which indicates the degree of influence of the single indicator on the emergency response capacity level. U indicates the obstacle degree of the single subsystem (first-level indicator), and the larger the value, the greater the obstacle effect of the indicator on the improvement of the emergency response capacity level in China.

### 3.5. Index System and Data Sources

**Figure 1:**
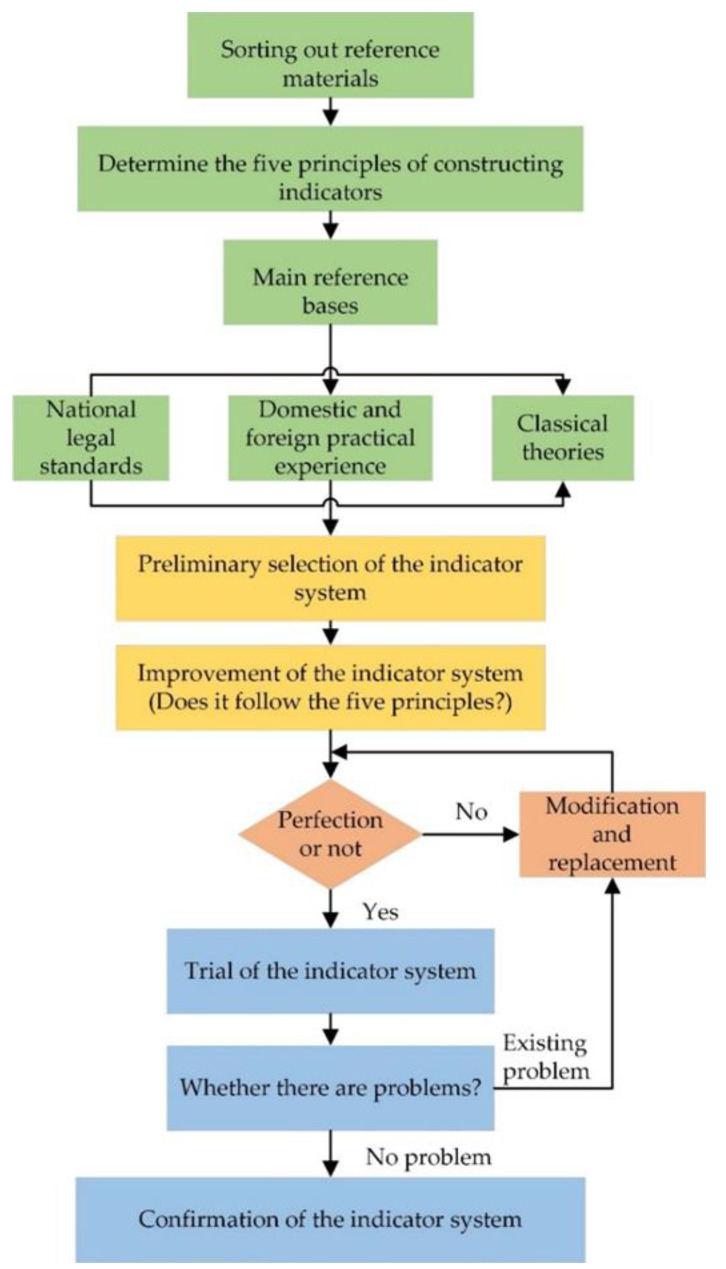
Flowchart of constructing emergency capability evaluation index system.

The evaluation index system is a combination set of two or more indexes that can effectively evaluate a specific system, with many functions such as evaluating the current situation, reflecting problems, and predicting trends [39]. Since the accuracy of indicators is the key to objectively and effectively evaluating the emergency response capacity of each province (city), its selection should follow five basic principles of systemic, feasibility, stability, coordination, and orientation, as well as reasonable construction methods [40,41]. The overall steps of index system construction in this study are divided into five parts: theoretical preparation, preliminary selection of index system, improvement of index system, trial of index system, and confirmation of index system, as shown in Figure 1.

#### 3.5.1. Construction of Indicators

**Figure 2:**
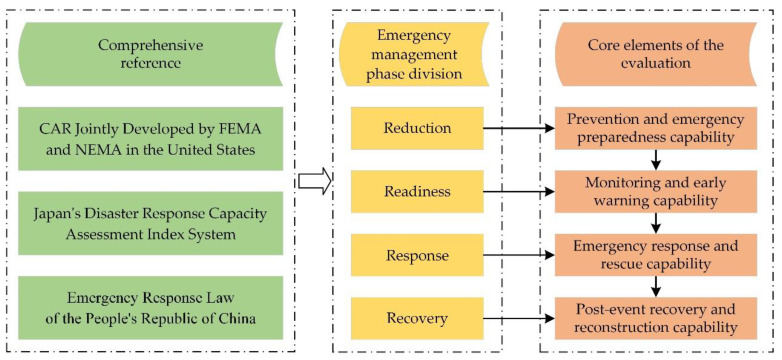
Emergency response capacity evaluation level 1 index framework.

Emergency management is a dynamic process, so the evaluation of emergency response capability should also be a whole process [42]. Based on the five basic principles mentioned above, this study starts from the concept of emergency management and comprehensively draws on FEMA’s Capability Assessment for Readiness (CAR) in the United States, Japan’s Disaster Emergency Response Capability Assessment Index System, China’s Emergency Response Law of the People’s Republic of China, and the Whole Process Theory of emergency management to form four primary indicators: prevention and emergency preparedness, monitoring and early warning capability, emergency response and rescue capability, and recovery and reconstruction capability, as shown in Figure 2.

The selection of the second-level index must form an interrelated and mutually constraining whole together with the first-level index and should fully reflect the focus of the first-level index on the basis of the number of control indicators. Prevention and emergency preparedness refers to the ability of the government to make the financial investment and careful design in terms of personnel, materials, and action plans in response to possible emergencies, and aims to lay the foundation for overall emergency management. In this study, seven indicators were selected for prevention and emergency preparedness, namely “the proportion of government financial expenditure for public security, the proportion of government financial expenditure for education, the proportion of government financial expenditure for transportation, the proportion of government financial expenditure for health care, the proportion of government financial expenditure for science and technology, the per capita gross regional product, and the number of students in ordinary colleges and universities per 10,000 people”. Monitoring and early warning capability refers to the capability of scientific monitoring and effective early warning for the direct purpose of minimizing losses before the occurrence of emergencies, which mainly includes forecasting and releasing the monitored information. Considering the representativeness and accessibility of the indicators, this study used “the number of universities and research institutes” as a proxy for “the emergency knowledge and skills training”. Finally, seven indicators were selected to evaluate and reflect the monitoring and early warning capability: television coverage, broadcast coverage, internet penetration rate, business volume of postal and telecommunication services per capita, number of universities and research institutes, number of social organization units, and percentage of illiterate population to total aged 15 and over. Emergency response and rescue capability refers to the ability to rescue and minimize the loss of emergency events through crisis communication and decision-making. In order to scientifically evaluate the emergency response and rescue capability, nine indicators were selected, including “number of health care institutions, number of beds in health institutions, hospital bed annual working days, health personnel per 10,000 people, the density of sewers in built districts, the daily disposal capacity of city sewage, number of public toilets per 10,000 people in cities, number of private cars per capita, and public recreational green space per capita”. Among them, “public recreational green space per capita” exists as an alternative indicator of “ number of emergency shelters”. Recovery and reconstruction capability refers to the ability to quickly restore the living environment, economy, work, and study to normal levels after the emergency event. In this study, seven indicators are used to accurately evaluate the recovery and reconstruction capability: the basic medical insurance participation rate, unemployment insurance participation rate, registered unemployment rate in urban area by region, proportion of labor force, proportion of government financial expenditure for social security and employment, general public budget revenue by region, and per capita disposable income of households. In summary, the index system constructed by this study contains four first-level indexes and 30 second-level indexes, as shown in Table 1.

#### 3.5.2. Data Source

**Figure 3:**
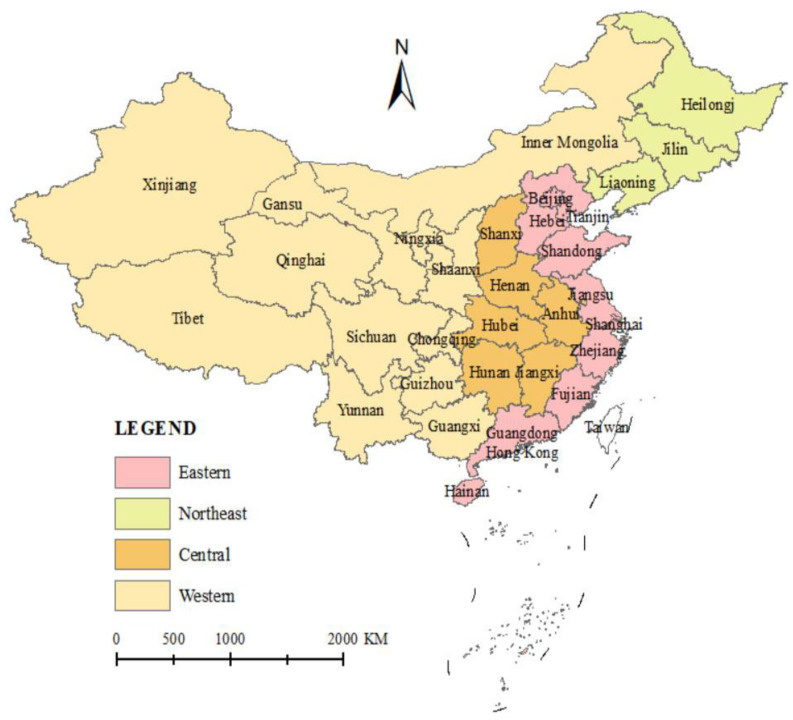
Distribution map of eastern, central, western regions and northeastern regions in China.

This study collects panel data for 31 provinces and municipalities in China (due to missing data, Taiwan, Hong Kong, and Macau are not included in the study for the time being) from 2011 to 2020, and the data are obtained from the China Statistical Yearbook and the China City Statistical Yearbook. A total of 310 observation samples are finally collated, the relevant economic data are processed to eliminate inflation, and individual missing data are supplemented by using the interpolation method. According to the division of administrative regions in mainland China, this study divides 31 provinces (cities) into four regions: eastern, central, western, and northeastern, and conducts a comprehensive assessment of the level of emergency response capacity in China as a whole and in the four regions to clarify their spatial and temporal distribution characteristics. The eastern region includes 10 provinces (cities) of Beijing, Tianjin, Hebei, Shanghai, Jiangsu, Zhejiang, Fujian, Shandong, Guangdong, and Hainan; the central region includes 6 provinces of Shanxi, Anhui, Jiangxi, Henan, Hubei, and Hunan; the western region includes 12 provinces (cities) of Sichuan, Chongqing, Guizhou, Yunnan, Tibet, Shaanxi, Gansu, Qinghai, Ningxia, Xinjiang, Guangxi, and Inner Mongolia; and the northeastern region includes three provinces of Heilongjiang, Jilin, and Liaoning, as shown in Figure 3. Considering the different measurement units of each evaluation index and the large difference in the range of values, this study standardized the original data so that the values were taken in the interval of [0, 1].

## 4. Results and Discussion

### 4.1. Temporal and Spatial Evolution of China’s Emergency Response Capacity

#### 4.1.1. Interannual Changes

**Figure 4:**
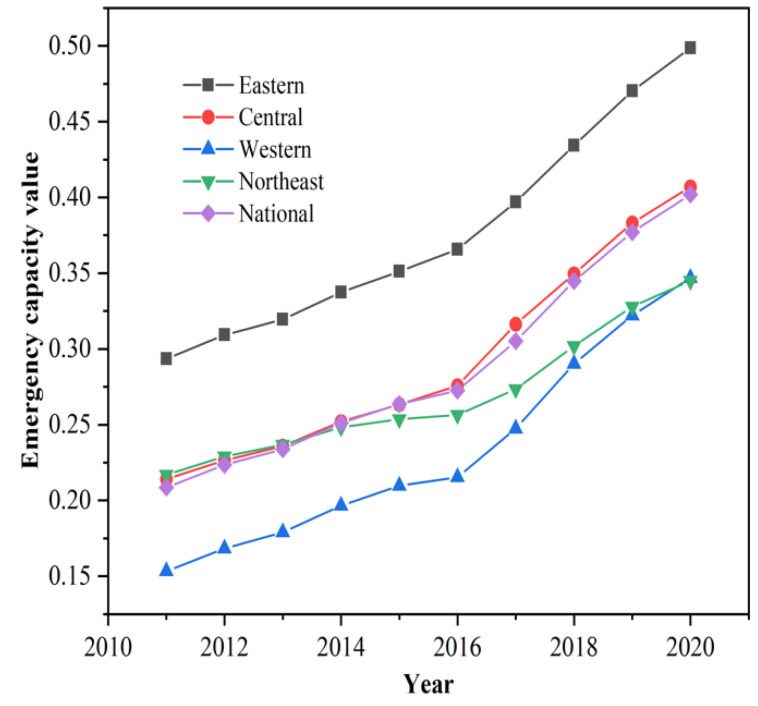
2011–2020 emergency response capacity levels in China and the four regions.

Figure 4 shows the interannual changes in the emergency response capacity of China and its four regions in the east, central, west, and northeast from 2011 to 2020. In terms of the overall national development trend, the average value of China’s emergency response capacity from 2011 to 2020 is 0.277, always showing a steady growth trend. Specifically, it rose from 0.204 in 2011 to 0.398 in 2020, an increase of 0.193 in 10 years, with an average annual increase of 9.46 percentage points, indicating that the overall trend of emergency response capacity development in China is better, and the provinces tend to complete the construction of functional systems for different stages of emergencies, strengthening the emergency response capacity for natural and man-made disasters, and showing a quality effect of a sudden increase in safety factor. This is due to the high importance the Chinese government has attached to the emergency management cause over the past 10 years and the successive introduction of policies and regulations represented by the Emergency Response Law of the People’s Republic of China in 2007, which has created many dividends for the improvement of China’s emergency response capability. The overall level has increased significantly in both 2017 and 2018, mainly because in December 2016, the CPC Central Committee and the State Council officially issued the Opinions on Promoting the Reform of Disaster Prevention, Reduction and Relief Systems and Mechanisms, which further clarified the new positioning, initiatives, and requirements for emergency management in the new era. From the four aspects of “improve the coordination system”, “improve the local management system”, “improve the social forces and market participation mechanism”, and “improve the overall disaster reduction capacity”, the reform of the emergency management system and mechanism has been comprehensively deployed, which has significantly improved the social emergency ability to resist natural disasters. In March 2018, the Ministry of Emergency Management of the People’s Republic of China was established, which not only realized the unification of emergency management objects, management responsibilities, and management processes at the system level, but also unified the terminology and technical standards of emergency functions of various departments at the technical level in all aspects of pre-, mid-, and post-event. All of these provide important strategic support and guarantee for the scientific policy-making, precise force, and efficient operation of China’s emergency management, and promote the continuous synergistic progress of emergency capability level.

As can be seen from Table 2, China’s emergency response and rescue capability dominate the overall emergency capability assessment, while the rank order of prevention and emergency preparedness capability, monitoring and early warning capability, and recovery and reconstruction capability shift significantly. From 2011 to 2020, monitoring and early warning capability increased from 0.033 to 0.120, and especially during the three years of 2018, 2019, and 2020, its rank order gradually rose from the bottom to the first, achieving a major leap of upgrading every year. This indicates that the monitoring and early warning of emergency management plays a pivotal role in the balanced development of comprehensive emergency response capabilities, and also reflects that China has realized a shift of the focus of work from “passive prevention—active response” to “active prevention—active response” after the establishment of the Ministry of Emergency Management in 2018. The rank order of emergency response and rescue capability consistently takes the first place until 2020, and then shifts to second place, with the rating increasing from 0.067 to 0.116, and its influence on comprehensive emergency response capability has weakened. The rank order of prevention and emergency preparedness capability has gradually changed from the second ranking in 2011 to the last ranking in 2020, with the rating increasing from 0.056 to 0.070, indicating that China’s emphasis on this aspect has gradually decreased and thus increased slowly in the past 10 years. The rank order of recovery and reconstruction capability has been fluctuating between the second and third positions, indicating that its influence on the balanced development of emergency response capability is relatively weak. Overall, China’s prevention and emergency preparedness, monitoring and early warning capabilities are lagging behind, and although they have been growing steadily in the last decade, they are still the shortcomings of the development of emergency response capabilities, and there is much room for improvement in the future.

#### 4.1.2. Interprovincial Changes

**Figure 5:**
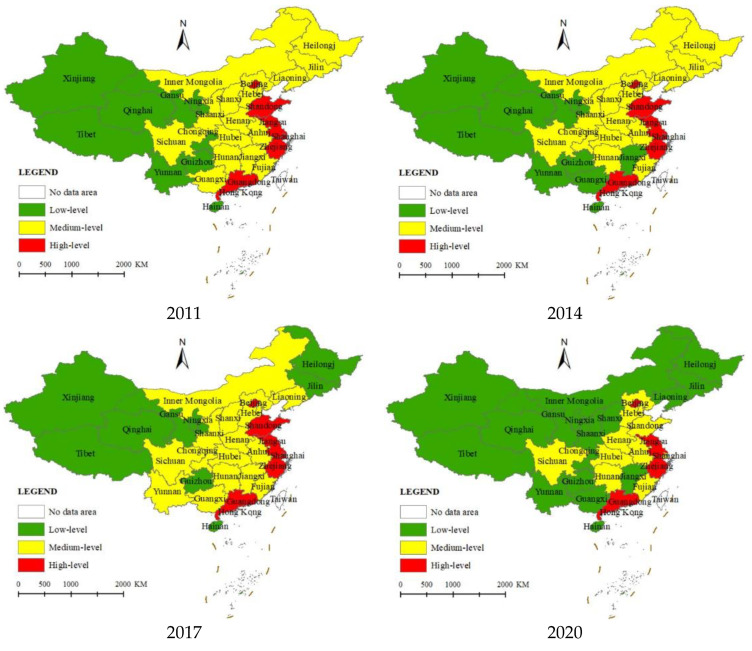
Interprovincial spatial distribution of emergency response capacity in China, 2011–2020.

Based on Jenks’ Natural Breaks method, the emergency response capacity levels of 31 provinces in China were classified into three levels: high level, medium level and low level. On this basis, Arcgis10.8 software is used to draw the spatial distribution map of China’s emergency response capacity in 2011, 2014, 2017 and 2020, as shown in Figure 5.

In 2011, China’s emergency response capability level showed an obvious aggregation effect of “the high-efficiency aggregation and the low-efficiency aggregation”. The provinces with high-level emergency response capability are concentrated in the eastern region, including Beijing, Shandong, Jiangsu, Shanghai, Zhejiang, and Guangdong, with scores ranging from 0.274 to 0.400, which is closely related to China’s policy of “shifting the economic center of gravity eastward” since the 1970s. The coastal areas have played a leading role in the economic transformation and upgrading of the country, and accordingly, the financial investment and policy pilots for the improvement of emergency response capacity are also far ahead in the country. The provinces with medium-level cover the whole central and northeastern regions as well as some western regions, with a total of 16 provinces, accounting for 52% of the total, including four in the western region. The provinces with medium emergency capacity cover the whole central, northeast, and some western regions, with a total of 16 provinces, accounting for 52%, including four in the western region. A total of nine provinces had a low emergency response capacity level: respectively, Xinjiang, Gansu, Ningxia, Qinghai, Tibet, Yunnan, Guizhou, Chongqing, and Hainan, all of which are concentrated in the western region (except Hainan). This is related to the aforementioned “Eastward Tilt policy”, where the rapid development of the eastern region absorbed a large number of laborers from the western region, leading to a prominent development imbalance and further widening the east–west gap. Although China proposed the “Western Development” policy in 2000, this gap still existed until 2011.

The overall emergency response capability level score range was 0.111–0.453 for Chinese emergencies in 2014, and although there was a steady increase compared with 2011, the distribution of high, medium, and low levels changed less than in 2011. The spatial distribution is consistent with that of 2011, except for Jiangxi and Guangxi, which decreased from medium level to low level, and Chongqing, which increased from low level to medium level. In 2017, the level of China’s emergency response capacity was in the range of 0.126–0.541, and the medium-level provinces were still concentrated in the central region. The obvious changes were that Heilongjiang and Jilin in the northeast region changed from the medium level in the year of 2014 to the low level, and some western regions such as Yunnan and Guangxi changed from the low level to the medium level. Due to the unbalanced development of China’s economy, the level of emergency response capacity in China in 2020 shows an obvious unbalanced trend of “high and medium level reduction and low-level expansion”, with the number of high- and medium-level provinces shrinking from 6 and 16 to 4 and 10, respectively. Among them, Shandong and Shanghai in the east have changed from high-level to medium-level provinces, Jilin in the northeast, Shanxi, and Jiangxi in the middle, and Shaanxi, Inner Mongolia, Guangxi, Yunnan, and Chongqing in the west have changed from medium-level to low-level provinces. Along with this shift, the number of low-level provinces increased to 17. In conclusion, the unevenness of China’s emergency response capacity remains a prominent problem, and there is a long way to go to deeply promote the reform of the emergency management system and emergency resource allocation.

### 4.2. Analysis of Regional Differences in China’s Emergency Response Capacity

Taking the emergency response capacity level score as the calculation index and bringing it into Equations (9) to (11), the Theil index of China’s inter-provincial emergency response capacity level from 2011 to 2020 was obtained and then its spatial and temporal distribution characteristics from the regional differences were further analyzed, and the results are shown in Table 3. From the calculation results, it can be seen that the regional differences in China’s emergency response capacity, except for the years 2015–2016, which showed a widening trend, have continued to decrease, from 0.0260 in 2011 to 0.0118 in 2020, a decrease of 54.6%. The largest decline in the Thiel index from 2017 to 2018, down 23.3% compared to 2017, was mainly due to the introduction of policy documents such as “Opinions of the State Council of the CPC Central Committee on Promoting Reform and Development in the Field of Work Safety” in December 2016, which clarified a series of reform measures and task requirements and indicated the directional path for the reform and development of the field of work safety in China, and greatly contributed to narrowing national regional differences and promote the coordinated development of each province by providing policy support. In addition, the comparison reveals that the intra-regional differences, inter-regional differences, and overall differences in China’s emergency response capacity are basically the same trend. From the source of regional differences, inter-regional differences show a reciprocating fluctuation of narrowing–expanding–narrowing from 2011 to 2020, but the overall trend is narrowing. The average contribution rate of inter-regional differences is 53.19%, which is higher than the contribution rate of intra-regional differences at 46.81%, and inter-regional differences become a decisive factor affecting the overall differences factor. This result indicates that although the contribution of inter-regional differences to the total differences tends to decrease, it is still an important reason affecting the coordinated development of regions, so the coordinated and sustainable development of inter-regional emergency resources is a key task that should be paid attention to in the future.

### 4.3. Spatial Correlation Analysis of China’s Emergency Response Capability

#### 4.3.1. Global Spatial Moran Index

To further explore the spatial agglomeration characteristics of emergency response capability level in China, based on Formula (12), the Global Moran’s I index value under the Queen’s weight matrix is calculated by using GeoDa software (Table 4). The calculated results show that the Moran’s I index and Z values are positive and all pass the significance test at the p < 0.05 level, indicating that the level of emergency response capacity in China is not completely randomly spatially distributed between 2011 and 2020, and there is a significant positive spatial autocorrelation, which is manifested by the provinces with high (or low) levels of emergency response capacity that tend to be relatively close to other provinces with high (or low) levels, i.e., they are spatially clustered. This also means that the level of emergency response capability of a province is not only influenced by its own environment, but also driven by the radiation of other surrounding provinces, the level of economic and scientific development, and the spatial layout of industries, and relevant emergency policies will further enhance the spatial correlation of development among provinces. 

From a numerical point of view, the Moran index fluctuated and declined from 0.356 in 2011 to 0.231 in 2020, reflecting a divergence in the spatial pattern of development of China’s emergency response capability level. The pattern of coordinated development in each region has not yet been formed, resulting in a series of problems such as the lack of effective connection of their development planning systems and that spatial management systems have not yet been formed, and although the Moran index in 2019 has increased from 0.210 to 0.231 in 2020 and the phenomenon of spatial agglomeration has been strengthened to a certain extent, the overall provincial cooperation system still needs to be improved, and there is still a long way to go to coordinate and promote the improvement of the national emergency response capability level.

#### 4.3.2. Local Spatial Moran Index

**Figure 6:**
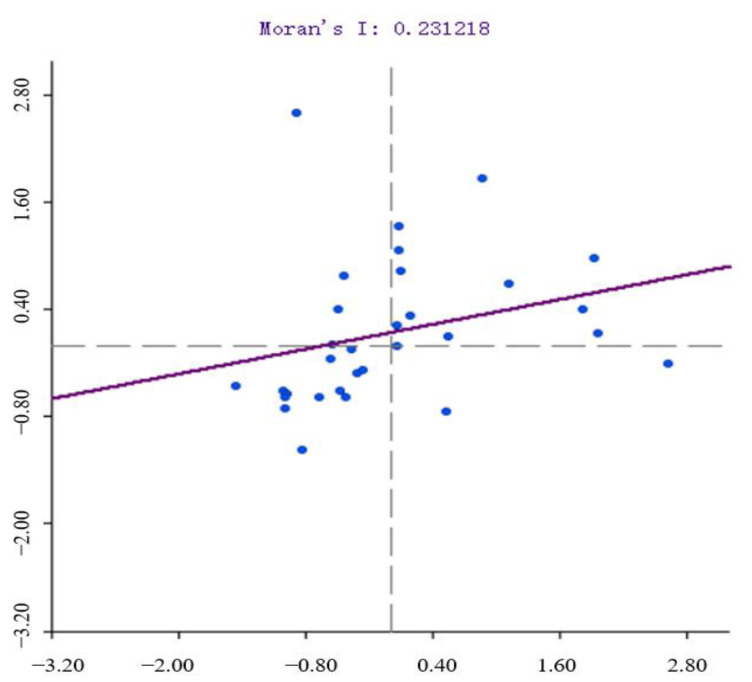
Moran scatter plot of emergency response capacity level in China in 2020.

The global Moran’s I index indicates that there is spatial agglomeration in the distribution of emergency response capability in China, but the overall spatial differences may mask the changes in local spatial differences, so the local autocorrelation model is introduced to further explore its spatial distribution characteristics. The Moran scatter plot of the spatially divergent states of China’s emergency response capacity level in 2020 was calculated using GeoDa software (Figure 6).

The slope of the blue line in the figure visualizes the increasing trend of Moran’s I index year by year, i.e., the overall spatial distribution tends to change from negative spatial autocorrelation to positive spatial autocorrelation. The four quadrants express four types of local spatial connections between a province and its surrounding provinces, namely “high-high (H-H)”, “low-low (L-L)”, “high-low (H-L)” and “low-high (L-H)”, and in order to visually reflect the specific quadrants into which each of China’s 31 provinces (cities) will fall in 2020, a dynamic distribution table is made (Table 5). According to the Moran scatter plot, it can be found that there is a significant spatial dependence on the level of emergency response capacity in China, and it shows the characteristics of the dual structure in space, i.e., the eastern provinces are mainly distributed in the “H-H” quadrant, and the western provinces are mainly distributed in the “L-L” quadrant. Specifically, 12 provinces (cities) are located in the “H-H” quadrant, 13 in the “L-L” quadrant, 2 in the “H-L” quadrant, and 4 in the “L-H” quadrant, accounting for 38.71%, 41.94%, 6.45%, and 12.90% of the total, respectively. This indicates that most of the provinces (cities) of China’s emergency response capacity are located in the “H-H” and “L-L” quadrants, and there is a positive spatial correlation.

**Figure 7:**
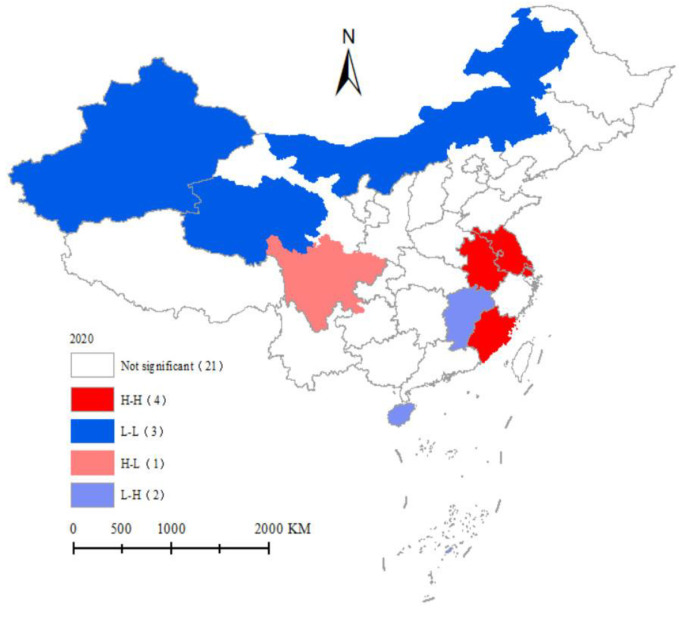
LISA cluster map of emergency response capacity level in China in 2020.

The local index of spatial connection is used to measure the degree of similarity (positive correlation) or difference (negative correlation) between the attributes of an observation unit and its surrounding units. Using GeoDA software, the LISA values of emergency response capability levels in different years were calculated for each province (city) in China, and the LISA distribution maps were plotted based on z-test (*p* ≤ 0.05) to understand more intuitively the geographical distribution of data. Due to the limitation of space, only the year 2020 is selected to show its spatial and temporal evolution characteristics (Figure 7): (1) The four provinces in the “H-H” quadrant are Shanghai, Jiangsu, Anhui and Fujian, mainly concentrated in the eastern region, which are more economically developed and have coordinated development, focusing on urban infrastructure construction while also strengthening external exchanges and cooperation, and forming agglomeration between provinces in the development process, with obvious spatial spillover effects. (2) Xinjiang, Qinghai, and Inner Mongolia belong to the “L-L” clustering pattern. These provinces are mainly located in the western region and have a certain degree of correlation with the surrounding areas, but their low level of aggregation, relatively backward economic and social development, and extensive resource exploitation methods make the role of regional growth poles insufficient, so the radiation and influence on the surrounding areas are also small. (3) The spatial heterogeneity of China’s emergency response capability level is significant, mainly in the “L-H” quadrant for Jiangxi and Hainan provinces and in the “H-L” quadrant for Sichuan province, showing negative spatial correlation. The analysis of the local Moran index shows that some provinces with high economic levels, convenient service facilities and transportation can improve the level of emergency response in the neighboring regions, which has a large “centripetal effect”; while in provinces with relatively backward and extensive development, the level of emergency response capacity in the neighboring regions is also low, which has a “centrifugal effect”. The interaction of these two effects finally forms the spatial clustering results of the emergency response capacity level in China.

### 4.4. Obstacle Factor Analysis

**Figure 8:**
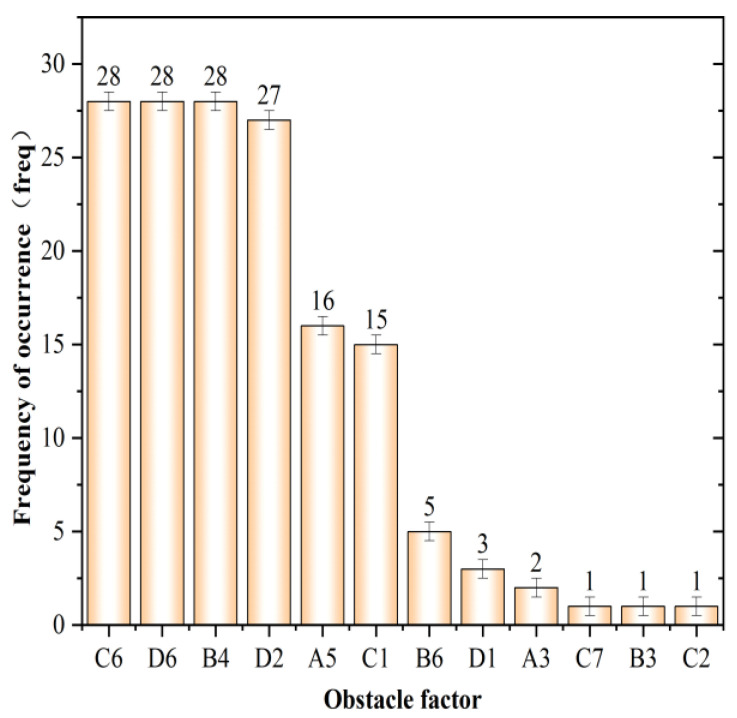
Frequency of occurrence of the top 5 obstacle factors in the emergency response capacity level.

The obstacle degree model was used to diagnose the obstacle factors affecting the emergency response capacity of 31 provinces in China in 2020, and the obstacle factors with an obstacle degree greater than 3% and ranked in the top five with obvious influence were selected according to the obstacle degree of individual indicators, as shown in Table 6 (due to the limitation of space, only the top three obstacle factors were analyzed in this study). On this basis, the frequency distribution histogram of 155 indicators ranked in the top 5 in 31 provinces was made, as shown in Figure 8.

Combining Table 6 and Figure 7, it can be seen that in the first obstacle factor, the main obstacle factor in Beijing, Shanghai, Zhejiang, Qinghai, and Ningxia is the “number of health care institutions”, which is mainly manifested by the fact that local health resources cannot meet the increasing demand for health services from residents. First, with the continuous improvement of China’s medical insurance system, the burden of residents’ medical expenses decreases, further releasing the demand for health services. Second, the pressure on health resources is increased by off-site medical treatment, for example, the number of medical and health institutions in Beijing in 2020 is 10,599, but its high-quality medical resources have to serve the residents of the whole country, resulting in the hospital bed annual working day of 223 days, and the demand for foreign patients to seek medical treatment in Beijing further exacerbates the contradiction between supply and demand of health resources. The main obstacle factor in Guangdong and Guizhou is the “unemployment insurance participation rate”, which is 28.54% in Guangdong and 7.72% in Guizhou, with corresponding obstacle degrees of 10.4% and 8.51%, respectively, both much higher than other indicators in the province. The obstacle factor of 24 regions (77.42%), such as Tianjin, Hebei, and Shanxi, etc. is the “business volume of postal and telecommunication services per capita”, which also reflects that China’s comprehensive communication capacity still has problems such as low quality of development and outstanding shortcomings, specifically as follows: (1) unbalanced supply of postal and telecommunication business in service objects and between subjects, especially postal business, 80% of its service objects are e-commerce, only 20% are traditional business and government affairs. (2) In the face of frequent natural disasters, there are still shortages in emergency communications and important communication guarantees. These problems constitute the biggest obstacle to improving the level of emergency response capability in most regions.

Among the second obstacle factors, the obstacle factor of 17 provinces (54.84%) such as Hebei, Jilin, and Liaoning is the “unemployment insurance participation rate”, which is mainly manifested as the imbalance in the supply and demand of labor resources and market when serious emergencies occur, thus inducing large-scale unemployment and social unrest. The occurrence of this phenomenon mainly includes the following two reasons: firstly, employees of township enterprises and individual entrepreneurs are not covered by unemployment insurance; secondly, peasant contract workers are not entitled to complete unemployment insurance. All these will lead to a surge of unemployed people after the emergencies, which will seriously affect the improvement of “Recovery and reconstruction capability”. The obstacle factor of nine provinces, including Beijing, Shanxi, and Inner Mongolia, is the “daily disposal capacity of city sewage”, indicating that 29.03% of the country’s regions have shortcomings in basic living facilities such as tap water supply, and there is a possibility of tap water interruption in cases of emergency. Guangdong’s basic medical insurance participation rate is only 87.07%, which is a big gap from the national average of 95.81% and the highest value of 99.9%, which also leads to it being the second most significant obstacle to the improvement of emergency response capacity, with an obstacle degree of 8.53%. In addition, the obstacle factor for Shanghai and Guizhou is the “business volume of postal and telecommunication services per capita”, with a barrier degree of 7.98% and 8%, respectively; the barrier factor for Tianjin and Hainan is the “number of health care institutions”, with a barrier degree of 9.62% and 7.95%, respectively.

In the third obstacle factor, the obstacle factor of nine provinces including Tianjin, Zhejiang, Shanxi, and Gansu is the “daily disposal capacity of city sewage”, and the average obstacle degree is 7.72%, which is consistent with the number of regions in the second obstacle factor. The lack of sewage treatment capacity is not only the third shortcoming in the improvement of emergency capacity, but also the bottleneck of urban development, which is inseparable from practical factors such as “backward traditional sewage treatment technology” and “excessive discharge of some enterprises” in most parts of China. The obstacle factor of Jilin, Heilongjiang, Hubei, and another seven provinces is the “general public budget revenue by region”, reflecting the problems of softening budget constraints, weakening the role of the budget, and hidden government debts in the emergency financial management of these regions. The obstacle factor of Xinjiang, Inner Mongolia, Liaoning, and another six provinces is the “proportion of government financial expenditure for science and technology”. Jiangsu is the “number of health care institutions”, Beijing and Shanghai are the “number of social organization units”, and Guangdong and Ningxia are the “business volume of postal and telecommunication services per capita”. It indicates that regions with a high level of emergency capability have higher requirements for emergency industry technology, industrial structure upgrading, urban informatization, and basic public medical facilities coverage, while regions with a relatively low level of emergency capability have yet to improve and enhance the degree of social organization development and emergency financial investment.

## 5. Conclusions

Scientific evaluation of emergency response capability is an indispensable and important measure to build a perfect national emergency system and improve the modern emergency management mode. In this paper, firstly, the Entropy Method is applied to measure the emergency response capability level of 31 Chinese provinces (cities) from 2011 to 2020 in four aspects: prevention and emergency preparedness, monitoring and early warning, emergency response and rescue, and recovery and reconstruction. Second, the Thiel index was used to explore the regional differences in China’s emergency response capacity and their root causes, and the contribution of inter-area differences and intra-area differences to the total differences were measured. Then, the Exploratory Spatial Data Analysis (ESDA) method was used to measure the spatial and temporal variation characteristics of emergency response capacity levels in each region. Finally, the obstacle factors and obstacle degree that affect the emergency response ability are explored by using the Obstacle Degree Model. The main conclusions are as follows (Table 7):

(1) The average value of China’s emergency response capacity during 2011–2020 is 0.277, and the overall trend has always shown steady growth. with an average annual growth rate of 9.46%. Among them, emergency response and rescue capabilities dominate and develop strongly in the overall emergency response capability assessment; the development of prevention and emergency preparedness capabilities, monitoring and early warning capabilities is relatively lagging behind. Although China has shifted its focus from “passive prevention—active response” to “active prevention—active response” after the establishment of the Ministry of Emergency Management of the People’s Republic of China in 2018, these two items are still shortcomings of the current development of emergency response capabilities, and there is much room for improvement in the future.

(2) The trend of emergency response capacity in the eastern, central, western, and northeastern regions from 2011 to 2020 is similar to that of the whole country, with the four regions showing a gradient of “high in the east, low in the west, and middle in the central and northeastern regions”. Among them, the gap between the emergency response capacity of the northeast region and the eastern and central regions has gradually expanded since 2014 and is equal to that of the western region in 2020.

(3) The level of emergency response capacity in China shows an obvious aggregation effect of “high-efficiency aggregation and low-efficiency aggregation” during 2011–2020. Due to the unbalanced development of China’s regional economy, the supply of emergency resources and the construction of emergency infrastructure in different provinces differ greatly, which leads to the unbalanced situation of “high- and medium-level reduction and low-level expansion” in 2020. Although China’s major decisions such as “Western Development”, “Regional Coordinated Development Strategy” and “The Belt and Road Initiative” have alleviated the development trend of polarization to a certain extent, the unevenness of emergency response capacity is still prominent, and there is a long way to go to further promote the reform of emergency management systems and emergency resource allocation.

(4) From the source of regional differences, the inter-regional differences show reciprocating fluctuation changes of narrowing–widening–narrowing from 2011 to 2020, but the overall trend is narrowing. Inter-regional differences are the decisive factor influencing the difference in emergency response capacity in China, with a mean value of 53.19%, higher than the contribution of intra-regional differences of 46.81%. The coordinated and sustainable development of inter-regional emergency resources is a key task that should be focused on in the future.

(5) In terms of spatial distribution characteristics, there is a significant spatial dependence in China’s emergency response capability level, and it shows a binary structure in space, i.e., the eastern provinces are mainly distributed in the “H-H” quadrant, while the western provinces are mainly distributed in the “L-L” quadrant. The interaction of “centripetal effect” and “centrifugal effect” finally formed the spatial clustering results of emergency response capability level in China.

(6) In terms of obstacle factors, the obstacle factor of 24 provinces (cities) such as Tianjin, Hebei, and Shanxi in 2020 is the “business volume of postal and telecommunication services per capita”, which is mainly manifested by the outstanding shortage of emergency communication capacity. The main obstacle factor for Beijing, Shanghai, Zhejiang, Qinghai, and Ningxia is the “number of health care institutions”, which mainly shows that local health resources cannot meet the growing demand of residents for health services. In Guangdong and Guizhou, the main obstacle factor is the “unemployment insurance participation rate”, and the corresponding obstacle degree is 10.4% and 8.51%, respectively, which is mainly manifested as the imbalance in the supply and demand of labor resources and market when serious emergencies occur, thus inducing large-scale unemployment and social unrest. In addition, indicators such as the “daily disposal capacity of city sewage”, the “general public budget revenue by region”, and the “proportion of government financial expenditure for science and technology” are also significant obstacles to the improvement of China’s emergency response capacity level. 

## 6. Policy Recommendations

The above research conclusions can provide the following policy recommendations for improving the level of emergency response capabilities in China (Table 8):

(1) The balance of the whole process of all phases of emergency management should be realized. The absence or weakening of any key stage in the whole process of emergency management may lead to a major failure in emergency management practice, resulting in irreversible casualties, property losses, or social disorder. In the face of the current shortcomings of China’s emergency management capabilities, which are “disposal-oriented and prevention-light”, prevention and emergency preparedness should be given priority, and major security risks should be prevented and resolved at the source, so that problems can be solved in the bud and before they become disasters. In particular, the government should improve the emergency planning system and integrate the risk control measures in the plan with socio-economic development, resource and environmental protection, and infrastructure construction to reflect the concept of “prevention-oriented” and “source management”. In addition, a sound forecasting and early warning system should be established according to the monitoring information of emergencies and risk assessment results, based on the degree of harm it may cause to determine the corresponding warning level, and to ensure that disaster information can be accurately and quickly conveyed to the public through a variety of channels such as television, radio, Internet, cell phones.

(2) Emergency coordination and balanced regional development should be strengthened. On the one hand, the regional coordinated development strategy and promote the reform of the emergency management system and emergency resource supply mechanism should be carried out. Taking into full consideration the interconnectedness between provinces (cities), break down the administrative barriers between regions by formulating reasonable emergency industry structure planning, policy guidance, and emergency technology innovation, while improving the top-level design of emergency management and industrial collaborative planning to promote the flow of national emergency resources as well as the optimization and upgrading of inter-regional emergency industry structure. On the other hand, the big cities radiate small cities and small cities drive small towns as the cooperation chain, and give full play to the spatial spillover effect with Beijing, Guangdong, Jiangsu, Zhejiang, and other provinces with high emergency response capacity levels as the leaders to form a mutually beneficial and complementary development pattern. At the same time, implement differentiated development strategies according to local conditions so that developed provinces can better play a radiation-driven role, and continue to strengthen the emergency industry to help the less-developed areas in the west and northeast, so as to efficiently promote the balanced development of the national emergency response capacity level.

(3) Make up for the shortcomings of emergency response capacity by applying precise measures. According to the analysis of obstacle factors, the main obstacles restricting the improvement of China’s emergency response capacity are “the business volume of postal and telecommunication services per capita”, “the daily disposal capacity of city sewage” and “the general public budget revenue by region”. Therefore, the following three aspects should be taken into account. First, establish a scientific and efficient emergency communication guarantee system. It can provide strong communication technology support for command and decision-making at all levels by building a special network for emergency communication and improving the emergency communication guarantee response mechanism for actual combat. Second, measures should be taken to strengthen the reserve management of emergency backup water resources, improve the monitoring and protection of water supply sources, and strengthen the maintenance and management of water supply pipeline networks to improve the emergency response capability of urban water supply. Third, improve the public security financial investment system. The national and local finances should make overall planning decisions and increase the financial investment in emergency medical and public security in areas with weak emergency response capacity in a focused and planned manner, so as to solve the problem of supply capacity for equalizing basic public security services in the western and northeastern regions.

## Figures and Tables

**Table 1:** Emergency response capacity evaluation index system and weight.

Target Layer	First-Level Index Layer	Weight	Second-Level Index Layer	Indicator Number	Weight
Emergency response capability level	Prevention and emergency preparedness capability (PEPC)	0.205	Proportion of government financial expenditure for public security (%)	A1	0.023
			Proportion of government financial expenditure for education (%)	A2	0.019
			Proportion of government financial expenditure for transportation (%)	A3	0.028
			Proportion of government financial expenditure for health care (%)	A4	0.018
			Proportion of government financial expenditure for science and technology (%)	A5	0.058
			Per capita gross regional product (yuan/person)	A6	0.037
			Number of students in ordinary colleges and universities per 10,000 people (person)	A7	0.021
	Monitoring and early warning capability (MEWC)	0.244	Television coverage (%)	B1	0.006
			Broadcast coverage (%)	B2	0.005
			Internet penetration rate (%)	B3	0.032
			Business volume of postal and telecommunication services per capita (yuan/person)	B4	0.114
			Number of universities and research institutes (pcs)	B5	0.031
			Number of social organization units (pcs)	B6	0.052
			Percentage of illiterate population to total aged 15 and over (%)	B7	0.004
	Emergency response and rescue capability (ERRC)	0.300	Number of health care institutions (pcs)	C1	0.062
			Number of beds in health institutions (bed)	C2	0.044
			Hospital bed annual working days (days)	C3	0.005
			Health personnel per 10,000 people (person)	C4	0.019
			Density of sewers in built districts(km/sq.km)	C5	0.028
			Daily disposal capacity of city sewage (10,000 cu.m)	C6	0.061
			Number of public toilets per 10,000 people in cities (unit)	C7	0.033
			Number of private cars per capita (unit)	C8	0.034
			Public recreational green space per capita (sq.m)	C9	0.015
	Recovery and reconstruction capability (RRC)	0.252	Basic medical insurance participation rate (%)	D1	0.041
			Unemployment insurance participation rate (%)	D2	0.061
			Registered unemployment rate in urban area by region (%)	D3	0.020
			Proportion of labor force (%)	D4	0.015
			Proportion of government financial expenditure for social security and employment (%)	D5	0.018
			General public budget revenue by region (100 million yuan)	D6	0.058
			Per capita disposable income of households (yuan)	D7	0.038

**Table 2:** Overall emergency response capability and subsystem scores in China from 2011 to 2020.

Year	Overall Emergency Capacity	Level I Index Emergency Capacity			
		PEPC	MEWC	ERRC	RRC
2011	0.2042	0.0559	0.0333	0.0674	0.0475
2012	0.2188	0.0578	0.0363	0.0710	0.0537
2013	0.2295	0.0591	0.0400	0.0778	0.0526
2014	0.2466	0.0622	0.0463	0.0828	0.0554
2015	0.2592	0.0622	0.0511	0.0872	0.0586
2016	0.2682	0.0626	0.0508	0.0919	0.0629
2017	0.3013	0.0649	0.0608	0.0971	0.0784
2018	0.3410	0.0663	0.0806	0.1033	0.0908
2019	0.3734	0.0666	0.1028	0.1102	0.0939
2020	0.3980	0.0696	0.1196	0.1159	0.0930
2011	0.2840	0.0627	0.0622	0.0904	0.0687

**Table 3:** The Theil index decomposition of regional differences and their sources of emergency response capacity in China from 2011 to 2020.

Year	Theil Index Decomposition				
	Total Regional Differences	Source of Differences		Contribution Rate (%)	
		Intra-Regional	Inter-Regional	Intra-Regional	Inter-Regional
2011	0.0260	0.0105	0.0155	40.46	59.54
2012	0.0243	0.0106	0.0137	43.57	56.43
2013	0.0233	0.0104	0.0129	44.76	55.24
2014	0.0214	0.0097	0.0117	45.47	54.53
2015	0.0210	0.0100	0.0110	47.67	52.33
2016	0.0218	0.0103	0.0115	47.32	52.68
2017	0.0189	0.0095	0.0093	50.62	49.38
2018	0.0145	0.0074	0.0070	51.43	48.57
2019	0.0133	0.0066	0.0067	49.73	50.27
2020	0.0118	0.0056	0.0063	47.12	52.88
Mean	0.0196	0.0091	0.0105	46.81	53.19

**Table 4:** Moran’s index of China’s emergency response capacity from 2011 to 2020.

Year	2011	2012	2013	2014	2015	2016	2017	2018	2019	2020
Moran’s *I*	0.356	0.327	0.288	0.271	0.227	0.253	0.240	0.221	0.210	0.231
z-value (variance)	3.610	3.282	2.925	2.784	2.364	2.623	2.531	2.443	2.275	2.555
*ρ*-value	0.010	0.010	0.010	0.010	0.010	0.010	0.010	0.010	0.020	0.010

**Table 5:** Quadrant distribution of China’s emergency capacity level in 2020.

Fall into the Quadrant	Province
H-H	Shanghai, Fujian, Tianjin, Anhui, Jiangsu, Shandong, Hebei, Hunan, Hubei, Henan, Zhejiang, Beijing
H-L	Guangdong, Sichuan
L-L	Chongqing, Guizhou, Shaanxi, Liaoning, Yunnan, Inner Mongolia, Jilin, Xinjiang, Qinghai, Ningxia, Heilongjiang, Gansu, Tibet
L-H	Hainan, Jiangxi, Guangxi, Shanxi

**Table 6:** The main obstacle factors and obstacle degrees in the indicator layer of emergency response capability level.

Region	1		2		3		4		5	
	Obstacle Factor	Obstacle Degree	Obstacle Factor	Obstacle Degree	Obstacle Factor	Obstacle Degree	Obstacle Factor	Obstacle Degree	Obstacle Factor	Obstacle Degree
Beijing	C1	0.1404	C6	0.1117	B6	0.1116	C2	0.0899	D6	0.0839
Tianjin	B4	0.0971	C1	0.0962	C6	0.0851	D6	0.0801	B6	0.0780
Hebei	B4	0.1497	D2	0.0913	A5	0.0789	C6	0.0727	D6	0.0658
Shanxi	B4	0.1295	C6	0.0742	D2	0.0736	D6	0.0675	A5	0.0668
Inner Mongolia	B4	0.1134	C6	0.0816	A5	0.0810	D2	0.0808	D6	0.0727
Liaoning	B4	0.1468	D2	0.0742	A5	0.0724	D6	0.0693	C1	0.0581
Jilin	B4	0.1285	D2	0.0768	D6	0.0750	A5	0.0715	C6	0.0709
Heilongjiang	B4	0.1460	D2	0.0727	D6	0.0691	A5	0.0687	C6	0.0652
Shanghai	C1	0.1112	B4	0.0798	B6	0.0793	C6	0.0780	D1	0.0755
Jiangsu	B4	0.1433	D2	0.1013	C1	0.0899	A3	0.0601	B3	0.0521
Zhejiang	C2	0.0931	D2	0.0920	C6	0.0825	D1	0.0735	D6	0.0617
Anhui	B4	0.1456	D2	0.0900	D6	0.0692	C6	0.0682	C1	0.0669
Fujian	B4	0.1165	B6	0.0800	D2	0.0794	D6	0.0713	C1	0.0695
Jiangxi	B4	0.1420	D2	0.0862	C6	0.0749	D6	0.0674	C1	0.0530
Shandong	B4	0.1959	D2	0.0973	A5	0.0627	C6	0.0571	D6	0.0546
Henan	B4	0.1606	D2	0.0977	C6	0.0692	D6	0.0673	A5	0.0600
Hubei	B4	0.1594	D2	0.0851	D6	0.0731	C6	0.0621	C1	0.0590
Hunan	B4	0.1426	D2	0.0867	D6	0.0686	C6	0.0666	A5	0.0511
Guangdong	D2	0.1040	D1	0.0853	B4	0.0796	A3	0.0760	C7	0.0737
Guangxi	B4	0.1208	D2	0.0825	D6	0.0717	A5	0.0706	C6	0.0600
Hainan	B4	0.0948	C1	0.0795	C6	0.0771	D6	0.0731	B6	0.0630
Chongqing	B4	0.1217	C6	0.0774	D6	0.0739	C1	0.0734	D2	0.0729
Sichuan	B4	0.1518	D2	0.0911	A5	0.0751	C6	0.0718	D6	0.0667
Guizhou	D2	0.0851	B4	0.0800	C6	0.0764	D6	0.0729	B6	0.0640
Yunnan	B4	0.0956	D2	0.0883	C6	0.0783	A5	0.0754	D6	0.0717
Tibet	B4	0.0848	C6	0.0734	D2	0.0729	C1	0.0725	D6	0.0706
Shaanxi	B4	0.1133	D2	0.0820	C6	0.0764	A5	0.0763	D6	0.0719
Gansu	B4	0.1084	D2	0.0780	C6	0.0748	D6	0.0719	A5	0.0701
Qinghai	C1	0.0783	C6	0.0778	D2	0.0774	D6	0.0753	A5	0.0732
Ningxia	C1	0.0823	C6	0.0783	B4	0.0776	D6	0.0766	D2	0.0698
Xinjiang	B4	0.0891	C6	0.0716	A5	0.0712	D6	0.0691	D2	0.0685

**Table 7:** Research conclusions.

**Research** **Conclusions**	Temporal and spatial evolution of China’s emergency response capacity	The average value from 2011 to 2020 is 0.277, with an average annual growth rate of 9.46%
		ERPC development is strong, PEPC and MEWC development is relatively lagging
		The four regions show a gradient of “high in the east, low in the west, and middle in the central and northeastern regions”
		The period 2011–2020 shows an obvious aggregation effect of “high-efficiency aggregation and low-efficiency aggregation”
		An unbalanced situation of “high- and medium-level reduction and low-level expansion” in 2020
	Analysis of regional differences in China’s emergency response capacity	The inter-regional differences show reciprocating fluctuation changes of narrowing-widening- narrowing from 2011 to 2020
		Inter-regional differences are the decisive factor influencing the difference in emergency response capacity in China, with a mean value of 53.19%
	Spatial correlation analysis of China’s emergency response capability	There is a significant spatial dependence in China’s emergency response capability level, and it shows a binary structure in space
		The eastern provinces are mainly distributed in the “H-H” quadrant, and the western provinces in the “L-L” quadrant
		The interaction of “centripetal effect” and “centrifugal effect” finally formed the spatial clustering results
	Analysis on the obstacle degree of China’s emergency response capability	The obstacle factor of 24 provinces (cities) such as Tianjin, Hebei, and Shanxi in 2020 is the “business volume of postal and telecommunication services per capita”, which is mainly manifested by the outstanding shortage of emergency communication capacity
		The main obstacle factor for Beijing, Shanghai, Zhejiang, Qinghai, and Ningxia in 2020 is the “number of health care institutions”, which mainly shows that local health resources cannot meet the growing demand of residents for health services
		The main obstacle factor for Guangdong and Guizhou in 2020 is the unemployment insurance participation rate, which is mainly manifested in the fact that large-scale unemployment and social unrest are easily induced when major emergencies occur

**Table 8:** Policy recommendations.

**Policy** **Recommendations**	Realize the whole process balance of all phases of emergency management	Prioritize prevention and emergency preparedness to resolve major security risks at the source
		Strengthen the management of emergency plans and integrate the risk control measures in the plans with socio-economic development, resource and environmental protection, and infrastructure construction
		Establish and improve the forecast and early warning system to ensure that disaster information is accurately and quickly transmitted to the public
	Emergency coordination and balanced regional development should be strengthened	In-depth implementation of the regional coordinated development strategy and promote the reform of the emergency management system and emergency resource supply mechanism
		Take big cities radiating small cities and small cities driving small towns as the cooperation chain to form a mutually beneficial and complementary development pattern
		Implementing differentiated development strategies according to local conditions, so that developed provinces can better play the role of radiation and drive
	Applying precise measures to make up for the shortcomings of emergency response capacity	Establishing a scientific and efficient emergency communication guarantee system
		Strengthening the management of emergency water reserves, improving the monitoring and protection of water supply sources, and strengthening the maintenance and management of water supply pipeline networks
		Improving the financial input system for public safety

## Data Availability

The evaluation data selected in this article are all derived from the “China Statistical Yearbook” (2011–2020) and the “China City Statistical Yearbook” (2011–2020).
